# When the meaningless make sense: Wordlikeness and affective norms for 4,800 pseudowords and 1,200 Spanish words

**DOI:** 10.3758/s13428-026-02976-4

**Published:** 2026-04-01

**Authors:** Celia Martínez-Tomás, Marc Guasch, Pilar Ferré, Miguel Lázaro, José Antonio Hinojosa

**Affiliations:** 1 Departamento de Psicología Experimental, Procesos Cognitivos y Logopedia, Universidad Complutense de Madrid, Madrid, Spain; 2 Instituto Pluridisciplinar, Universidad Complutense de Madrid, Madrid, Spain; 3 Department of Psychology and CRAMC, Universitat Rovira I Virgili, Tarragona, Spain; 4 Centro de Investigación Nebrija en Cognición (CINC), Universidad Nebrija, Madrid, Spain

**Keywords:** Pseudowords, Valence, Arousal, Wordlikeness, Morphology

## Abstract

Most research using pseudowords has focused on the contribution of sublexical properties to the study of word processing. However, recent evidence suggests that pseudowords can also provide insights into the semantic and emotional aspects of language processing. Research in this field would greatly benefit from datasets providing estimations for pseudoword stimuli in the various lexicosemantic variables that have been shown to affect word recognition. Such datasets are currently lacking. In the present study, we introduce normative data for 4,800 pseudowords and 1,200 Spanish words, which were rated by 1,210 participants on three dimensions: wordlikeness, valence, and arousal. The stimuli were derived from emotional and neutral base words, and the morphological structure of the pseudowords was manipulated to create four versions combining real roots and suffixes with non-roots and non-suffixes. Additionally, we computed the normalized Levenshtein distance, the number of orthographic neighbors, and the mean Levenshtein distance to the 20 closest orthographic neighbors (OLD20) to examine the influence of objective measures on perceived wordlikeness. The results showed that the morphological structure of the pseudowords had a gradual effect on wordlikeness, valence, and arousal scores, with those combining real roots and suffixes being rated the highest. Furthermore, affective variables were found to consistently predict perceived wordlikeness ratings, whereas objective measures only accounted for a small proportion of the variance. This empirically validated set of well-controlled pseudowords is a valuable resource for researchers interested in the effects of morphology and affect on word processing. The complete database can be downloaded from: https://osf.io/baues/.

**Supplementary Information:**

The online version contains supplementary material available at 10.3758/s13428-026-02976-4.

## Introduction

Research involving pseudowords—sequences of letters that adhere to the phonotactic and orthographic rules of a given language yet are not found in the mental lexicon—has made valuable contributions to our understanding of language comprehension and production. For example, pseudowords have deepened our understanding of letter identification and coding during orthographic processing (Grainger, 2018; Lupker et al., 2008; Perea et al., 2022, 2023), the sublexical constraints involved in grapheme-to-phoneme conversion rules (Kwok et al., 2017; Perea & Estévez, 2008), and sound-symbolic effects that index nonarbitrary mappings between forms and meanings (Schmidtke & Conrad, 2024; Sidhu & Pexman, 2018). Pseudowords have also provided valuable insights into both lexical and supralexical processes. These include acquiring novel meanings (Guasch & Ferré, 2021; Rodríguez-Gómez et al., 2018), as well as making linguistic predictions about grammatical categories (Bonhage et al., 2015) and about agreement relationships based on morphosyntactic features (e.g., gender, number, or person; Franck & Wagers, 2020). This has been partially achieved through the use of “Jabberwocky” sentences, in which content words are replaced by pseudowords but morphological markers and function words are retained.

One important line of research with pseudowords has focused on wordlikeness. This concept has been delineated as the extent to which a sequence of phonemes is characteristic of words within a given language (Bailey & Hahn, 2001), or the measure of the probability that a given string of letters could constitute a valid or plausible word in a specific language (Frisch et al., 2000). Based on these notions, wordlikeness has been measured through rating tasks that used Likert scales to collect subjective estimations of the probability that nonwords are existing words (e.g., Aljasser et al., 2025; Frisch et al., 2000; Vitevitch & Donoso, 2012). Alternatively, several objective measures have been proposed to account for likelihood of a string of letters being a word. Common objective measures include normalized Levenshtein distance (i.e., the number of insertions, deletions, or substitutions required to transform a nonword into a genuine word, NLD), the orthographic and phonological lexical neighbors (i.e., the number of words that can be formed by substituting a single letter in a nonword of equal length, N), orthographical Levenshtein distance 20 (i.e., the mean Levenshtein distance to the 20 closest orthographic neighbors of the nonword, OLD20), or phonotactic/orthographic sequence probability (i.e., the position-specific frequency of phonemes and sequences of phonemes) (Coltheart et al., 1977; Duchon et al., 2013; Ellis & Beaton, 1993; Keuleers & Brysbaert, 2010; Levenshtein, 1966; Yarkoni et al., 2008). Overall, pseudowords with higher wordlikeness are identified faster in recognition tasks and recalled more easily in memory tasks (Janse & Newman, 2013; Thorn & Frankish, 2005; Vitevitch & Luce, 1999; Vitevitch et al., 1997).

Prior research has identified phonotactic/orthotactic probability (i.e., the frequency of the possible sequential arrangements of sounds) and/or the similarity between a string of letters and particular words in the lexicon as two of the main sources contributing to wordlikeness (Bailey et al., 2001; Frisch et al., 2000; Lipinski & Gupta, 2005; Vitevitch & Luce, 1999, 2016). Notably, the morphological structure of a pseudoword also influences the likelihood of a string of letters being considered an existing word (Beyersmann et al., 2020; Carota et al., 2016; Lázaro et al., 2015). For instance, evidence from lexical decision studies indicates that complex pseudowords (i.e., those comprising a stem and a suffix) elicit delayed rejection times relative to those stimuli without morphological constituents (Burani et al., 1999; Dawson et al., 2018; Lázaro et al., 2022). Also, masked and cross-modal priming studies have shown that suffixed versus non-suffixed pseudoword primes accelerate the visual identification of a stem target (Longtin & Meunier, 2005; Morris et al., 2011).

Another fruitful area of research has examined the role of affective dimensions such as valence (i.e., the hedonic tone of a word, ranging from negative to positive) and arousal (i.e., the degree of activation elicited by a word, ranging from calming to exciting) in pseudoword processing. Current data from lexical decision tasks suggest that pseudowords derived from emotionally intense base words are categorized more slowly than those derived from neutral words (Gatti et al., 2023; Sulpizio et al., 2021). Also, studies pairing pseudowords with emotional stimuli, such as faces (Gu et al., 2023), odors (Speed et al., 2021), and pictures (Rummer & Schweppe, 2019), have examined the acquisition of emotional meaning. The evidence summarized here suggests that pseudowords are useful stimuli for exploring all levels of language processing.

Studies in this area have benefited from tools that enable the generation of meaningless stimuli while preserving the formal properties of a language. Pseudoword generators such as Wuggy (Keuleers & Brysbaert, 2010), WordGen (Duyck et al., 2004), the ARC Nonword Database (Rastle et al., 2002), the Character-Gram Chaining Algorithm (CGCA, König et al., 2020), and UniPseudo (New et al., 2024) can produce pseudowords with structural features similar to those of real words (e.g., syllables, affixes, onsets, nuclei, codas, rhymes) in languages such as Dutch, English, German, French, and Spanish. These tools also enable the control of sublexical properties, including orthographic neighborhood size, orthographic similarity, and bigram and trigram frequency, as well as letter and syllable length. Using stimuli created by these generators has increased our understanding of the role of sublexical features in word processing (see Martínez-Tomás et al., 2025, for a review). However, they are less effective at controlling for the lexicosemantic features or for the morphological structure of pseudowords. One way to overcome this limitation is to collect scores from a large number of participants for a large set of stimuli across various psycholinguistic variables. To date, this type of study has only been conducted with words, providing data on variables like concreteness (Brysbaert et al., 2014a, 2014b; Ćoso et al., 2019), subjective age of acquisition (Alonso et al., 2015; Citron et al., 2014), valence and arousal (Moors et al., 2013; Warriner et al., 2013), or primary emotions such as anger, fear, sadness, awe, relief, or happiness (Ćoso et al., 2023; Hinojosa et al., 2024). However, normative studies collecting data on lexicosemantic features of pseudowords could be particularly useful for shifting the focus from using pseudowords to study sublexical processes such as orthographic, acoustic, and phonological ones. This would contribute to expanding our understanding of lexical processes such as morphological composition (e.g., Dawson et al., 2018), meaning acquisition (e.g., Elgort et al., 2024), or the activation and representation of conceptual features such as concreteness (e.g., Mestres-Missé et al., 2014) and valence (e.g., Sulpizio et al., 2021). These datasets could be also useful for studying the interaction between sublexical and lexical features, as in research on sound symbolism (Schmidtke & Conrad, 2024) and orthographic-to-meaning consistency (Yap et al., 2015).

To fill in this gap, in the current study we selected a pool of 1,200 Spanish nouns with different emotional connotations (i.e., positive, negative, and neutral), as pseudowords are increasingly being used to examine issues such as the acquisition or the processing of emotional meaning (Gu et al., 2021; Kuchinke & Mueller, 2019; Mestres-Missé et al., 2014). Prior research has underscored the importance of morphological structure in pseudoword research (Beyersmann et al., 2020; Dawson et al., 2018). Therefore, we generated four versions of each base word by altering its morphological composition. The Spanish linguistic morphology employs roots and affixes as elementary structural components in word formation (Varela, 1990). Roots provide the core lexical meaning of words, while affixes are bound morphemes that are attached to roots to modify or supplement their meanings. These can be used as prefixes (i.e., they are placed before the root, as the prefix “sub-” in *sub-título*; subtitle) or suffixes (i.e., they are placed after the root, as the suffix “-ista” in *deport-ista*, athletic). Since suffixes are often used to convey inflectional or derivational meaning in Spanish, our focus was on these affixes. Prefixed words were avoided because their semantic domains are much narrower than those of suffixes. Crucially, they often indicate negation or intensity, which could introduce an artificial bias into valence and arousal scores. For instance the prefix “in-” in the word *infeliz* (unhappy) reverses the positive valence of the unprefixed word *feliz* (happy). Also, their influence on the words they are attached to differs from that of suffixes (e.g., Beyersmann et al., 2015; Giraudo et al., 2025). Consequently, in the first version, pseudowords retained the root of the base word (e.g., *delicad-* in the word *delicadeza,* delicacy) and were affixed with a real suffix (i.e., a suffix that exists in Spanish, e.g., -*ura* in the pseudoword *delicadura*). The second version contained the root of the base word and a non-suffix ending, which is a real suffix with letter transpositions (e.g., *delicadrua*). The third version involved forming pseudowords from a non-root (i.e., the root of the corresponding base word with letter transpositions) and the suffix of the base word (e.g*., dadeliceza*). The fourth version contained the non-root from the third version and the non-suffix ending from the second version (e.g., *dadelicrua*).

Apart from collecting ratings of affective variables (i.e., valence and arousal), and given the importance of wordlikeness in pseudoword research (Crepaldi et al., 2010; Lázaro et al., 2024; Longtin & Meunier, 2005), we collected scores for this variable as an indicator of the perceived probability of a sequence of letters being an existing word (Frisch et al., 2000; Vitevitch & Donoso, 2012). Additionally, we examined the relationship between subjective estimations for wordlikeness and several objective measures for quantifying wordlikeness based on string similarity computed as the distance either to the corresponding base word (NLD) or to the whole lexicon (N and OLD 20).

Furthermore, we explored the relationship between the morphological structure of pseudowords and their wordlikeness, as well as their valence and arousal. This seems particularly promising in light of recent evidence suggesting that morphology plays a role in conveying affective meaning (Hinojosa et al., 2022). Based on prior evidence (Dawson et al., 2018; Lázaro et al., 2023), we expected that pseudowords that included both roots and suffixes would be scored higher in wordlikeness, valence, and arousal than pseudowords without morphological constituents.

## Method

### Participants

Ratings were collected from 1,210 native Spanish individuals (949 female, 253 male, and eight who preferred not to answer). Their mean age was 24.15 years (standard deviation [*SD*] = 9.21), ranging from 18 to 67 years. The sample size calculation was based on that used in previous normative studies, which typically collected 10–30 responses per questionnaire (e.g., Guasch et al., 2016; Speed & Brysbaert, 2024; Stadthagen-Gonzalez et al., 2017; Warriner et al., 2013). The average number of observations per word and variable was 26.72 (*SD* = 1.83; range = [25–34]). The mean reliability of the questionnaires was higher than 0.82 for all variables (see the Results section). Most of the participants were students from various universities across Spain: Universidad Complutense de Madrid (UCM, 48.93%), Universidad Nacional de Educación a Distancia (UNED, 10.25%), Universidad Rey Juan Carlos (URJC, 8.93%), Universitat Rovira i Virgili (URV, 8.68%), Universidad de Murcia (UM, 6.03%), Universidad Pontificia de Salamanca (UPSA, 4.71%), and others (8.02%). Additionally, there were non-university participants (4.46%). Participants either volunteered or took part in exchange for extra course credits. All participants signed an informed consent form before completing the questionnaires. The protocol was approved by the Psychological Ethics Committee of the Universidad Complutense of Madrid, and the study was conducted in accordance with the Declaration of Helsinki.

### Materials

First, we selected a set of 1,200 positive (valence ratings ≥ 6), negative (valence ratings ≤ 4), and neutral (valence ratings 4 − 6) nouns without prefixes from several normative studies (Ferré et al., 2012; Guasch et al., 2016; Hinojosa et al., 2016, Stadthagen-Gonzalez et al., 2017) using emoFinder (Fraga et al., 2018). Of the 1,200 words, 1,127 had different stems. In a few cases, the same stem appeared in two words (e.g., *vibrador*, “vibrator,” and *vibración*, “vibration”). The words also contained 91 different derivational suffixes—108 if the masculine and feminine forms are considered, as in *jugadora* (“feminine player”) and *domador* (“masculine tamer”). Secondly, as current pseudoword generators do not permit highly constrained control over the morphological structure of pseudowords, we identified the roots and suffixes of each word. Four experimental conditions of pseudoword were then created from each of these words (see Table 1). All pseudowords met the orthographic and phonological rules of Spanish, and were between 5 and 12 letters long. The number of letters in the suffixes and non-suffixes was the same as that in the suffix of the base word, and the non-roots and non-suffixes were created by transposing the letters of the roots and suffixes of real words. The four versions were as follows:

1. Root + Suffix (R + S): The root of the base word was combined with a different genuine derivative suffix.
2. Root + Non-Suffix (R + NonS): A non-suffix was added to the root of the base word. The non-suffix was formed by transposing the letter from the suffix used in the R + S condition.
3. Non-root + Suffix (NonR + S): The suffix of the base word was attached to a non-root. The non-root was formed by transposing letters from the root of the base word.
4. Non-root + Non-Suffix (NonR + NonS): non-roots from the third condition were combined with a non-suffix from the second condition.

**Table 1:** Examples of the four experimental conditions of pseudowords

Word	Root	Suffix	R + S	R + NonS	NonR + S	NonR + NonS
comparación (*comparison*)	compar-	-ación	compar + adera	compar + erada	cromap + ación	cromap + erada
relojero (*clockmaker*)	reloj-	-ero	reloj + ado	reloj + oda	jerol + ero	jerol + oda

### Procedure

Words and pseudowords were rated through an online questionnaire in the form of a website created from scratch. The 6,000 stimuli (1,200 base words and 4,800 pseudowords) were pseudo-randomly divided into 25 questionnaires, each containing 240 stimuli for the three variables: wordlikeness, valence, and arousal. In total, 75 questionnaires were created. Each list included 48 stimuli from each of the five conditions, with the only constraint being that base words and their corresponding R + S, R + NonS, NonR + S, and NonR + NonS pseudowords were assigned to different questionnaires.

**Fig. 1:**
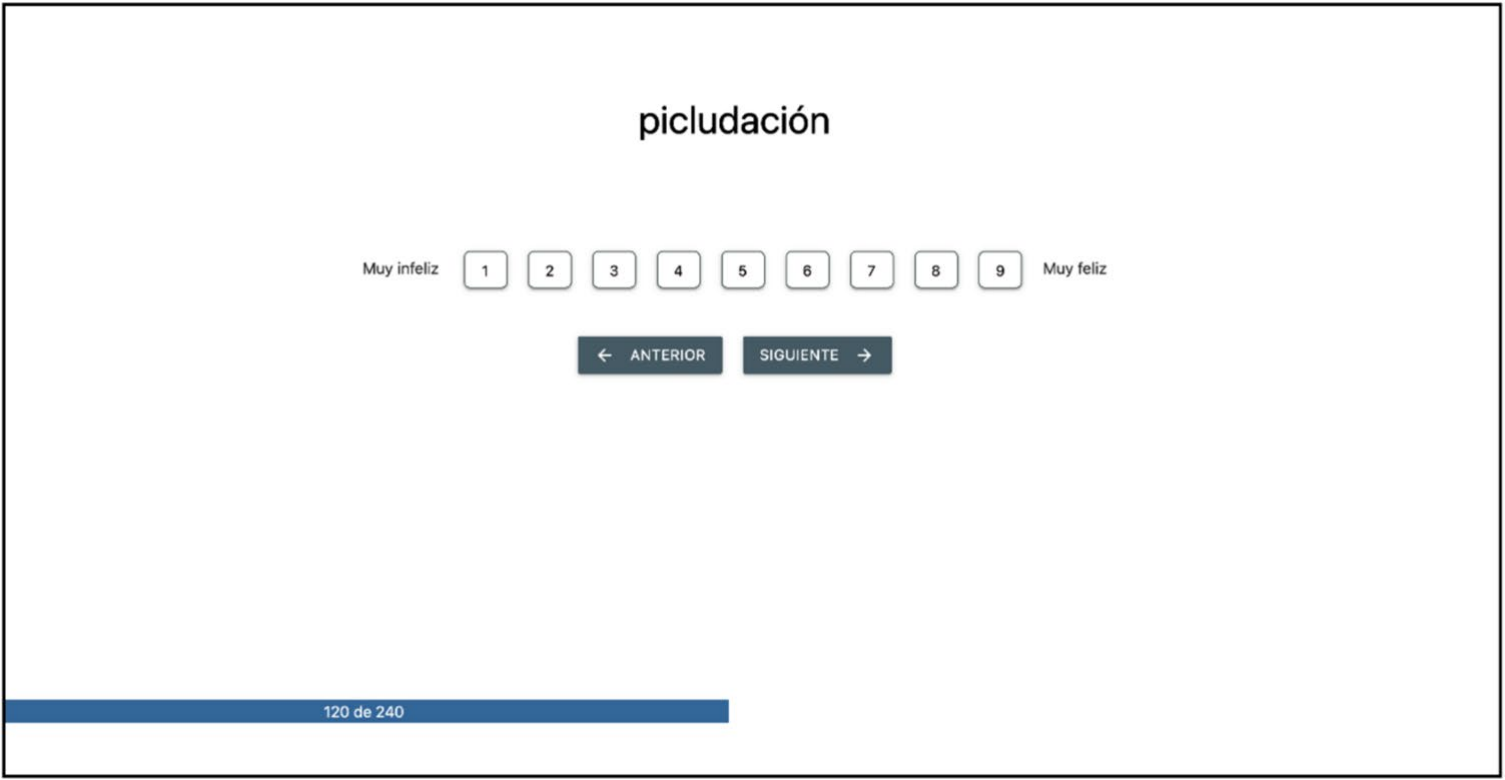
Layout of the rating screen for the pseudoword *picludación* and the variable *valencia* (*valence*)

On the first page, participants were given information about the purpose of the study, along with a confidentiality statement. They were then asked to provide demographic information concerning their age, gender, and university. On the next screen, they were given instructions for scoring the stimuli according to the randomly assigned variable. The same instructions as those used by Frisch et al. (2000) for wordlikeness were used, with participants rating each stimulus on a scale from 1 to 7 (1 = very unlikely that the stimulus could be a word in Spanish; 7 = very likely that the stimulus could be a word in Spanish). Regarding the affective variables, we used the same instructions and scale for valence and arousal as Stadthagen-Gonzalez et al. (2017). Participants rated valence and arousal on a scale from 1 to 9 for each stimulus (1 = very unhappy, 9 = very happy for valence, and 1 = very calm, 9 = very active for arousal). The full instructions given to participants can be found in the Appendix. After receiving the instructions, participants began scoring the stimuli, which appeared one by one in the center of the screen (see Fig. 1). Participants used the scale under each stimulus to complete their ratings. After the participant had rated a stimulus, the screen automatically moved on to the next one. However, there were “next” and “previous” buttons, so participants could go back and forth to modify their answers if they wished. Rating each stimulus was mandatory in order to proceed to the next one. Participants were randomly assigned to questionnaires and could complete more than one (mean = 1.94, *SD* = 1.09). However, to prevent potential response biases (e.g., higher wordlikeness scores after rating the same stimuli in terms of valence), participants were never asked to rate the same stimuli twice, even when different variables were considered. Reaction times were not collected and there were no time constraints for the rating process. Participants took approximately 20 min to complete a questionnaire.

### Data trimming and description of the dataset

A total of 2,348 questionnaires were completed. These were submitted to a trimming procedure to exclude participants who responded with anomalous patterns. In particular, we discarded questionnaires that were clearly filled out carelessly (e.g., where almost all the answers were the same value, accounting for 0.26% of the data), and those where the ratings correlated with the mean rating of the same stimuli in the other questionnaires of the same variable by less than 0.10 (accounting for 14.40% of the data). This measure enabled us to detect participants who had misunderstood the scales or responded randomly. As a result of the exclusion criteria, 344 questionnaires were removed (14.65% of the total): 23 wordlikeness questionnaires, 47 valence questionnaires, and 274 arousal questionnaires. Thus, 2,004 questionnaires were analyzed, corresponding to 1,094 different participants. The final database is available at https://osf.io/baues/ It takes the form of three CSV files, one for each variable, which include the following information for each word and pseudoword: mean rating, standard deviation, and number of raters. Furthermore, in line with Taylor et al. (2023a, 2023b), the raw trial-level data before trimming is also included. One file contains information about participants and another contains all the responses.

## Results

### Reliability and validity of the norms

First, we examined the reliability of the data using a split-half procedure with the Spearman–Brown correction in the *multicon* package (Sherman, 2015) in R (R Core Team, 2024). We performed 100 simulations for each of the 25 questionnaires and for each of the three variables. The average reliability for each variable was as follows: *r* =.97 [.97–.98] for wordlikeness; *r* =.90 [.86–.92] for valence; and *r* =.82 [.74–.91] for arousal.

To examine the validity of the questionnaires, we focused on the words and the valence and arousal variables. However, we could not examine the validity of wordlikeness, valence, and arousal scores for pseudowords, as there are no prior normative studies on these stimuli in Spanish. We used emoFinder (Fraga et al., 2018) to obtain valence and arousal ratings for 1,060 words. These data were obtained from the databases of Stadthagen-González et al. (2017; 1,027 words), Guasch et al. (2016; 18 words), Ferré et al. (2012; 10 words), and Hinojosa et al.(2016; five words). No data on these variables were available for 140 of the words. The correlations were *r* =.93, *p* <.001 for valence and *r* =.75, *p* <.001 for arousal.

### Wordlikeness

**Fig. 2:**
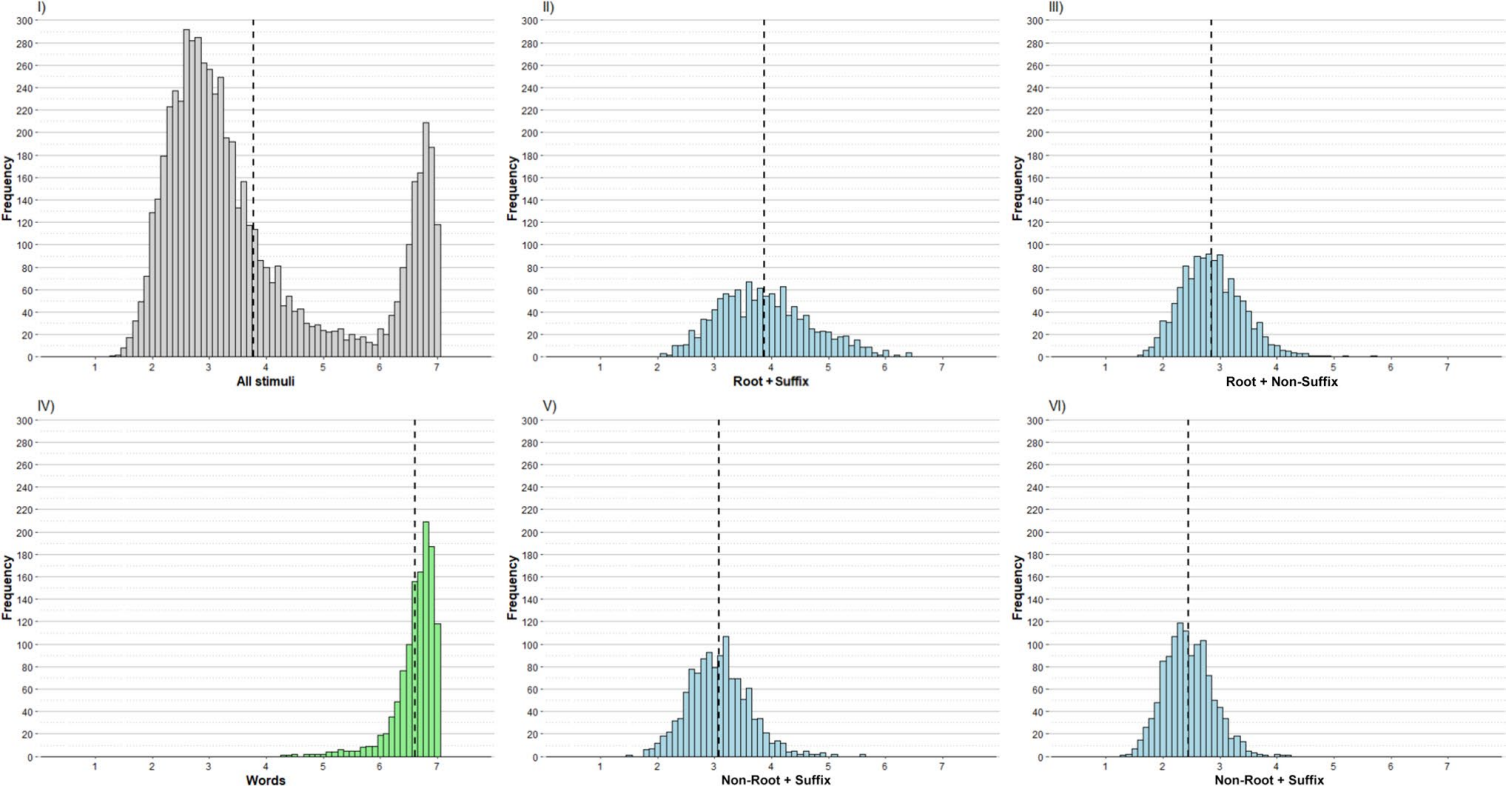
The histograms show wordlikeness for the full set of stimuli (panel I), the four types of pseudowords (R + S, panel II; R + NonS, panel III; NonR + S, panel V; NonR + NonS, panel VI), and the words (panel IV). The dashed vertical lines indicate the means. The mean wordlikeness score was higher for the R + S pseudoword condition, and decreased gradually for the NonR + S, R + NonS, and NonR + NonS conditions

First, we examined the distribution of ratings for wordlikeness. Figure 2 shows the histograms for all the stimuli, the words, and each type of pseudoword.

The distribution of ratings across the 6,000 stimuli (see panel I) is bimodal, with peaks near the top and bottom of the 1–7 scale. The overall mean is 3.77 (*SD* = 1.60). However, examining the distribution of wordlikeness for words only (panel IV) reveals that the upper-end peak is almost entirely due to words, which have an average rating of 6.60 (*SD* = 0.38) and a minimum value of 4.28—in other words, all words are above the midpoint of the scale.

The four graphs (panels II, III, V, and VI) show that the distribution of pseudowords is approximately normal. However, there are clear differences in terms of mean and range (see Table 2).

**Table 2:** Descriptive statistics for wordlikeness across types of stimuli

Type of stimuli	Mean	*SD*	Interval	Range
Words	6.60	0.38	[4.28, 7.00]	2.72
Root + Suffix (R + S)	3.88	0.82	[2.07, 6.42]	4.35
Root + Non-Suffix (R + NonS)	2.86	0.54	[1.60, 5.68]	4.08
Non-Root + Suffix (NonR + S)	3.07	0.55	[1.46, 5.60]	4.14
Non-Root + Non-Suffix (NonR + NonS)	2.44	0.42	[1.32, 4.25]	2.93
**Full set**	**3.77**	**1.60**	**[1.32, 7.00]**	**5.68**

We used a one-way analysis of variance (ANOVA) to compare the wordlikeness ratings of the four pseudoword conditions. There were differences between them, *F*(3, 4,796) = 1,216.03, *p* <.001, *η*^2^_p_ =.43. Bonferroni-corrected paired comparisons revealed that all differences were significant (all *p*s <.001). The pseudoword condition with the highest mean (and also the widest range of ratings) was R + S, followed by NonR + S, R + NonS, and NonR + NonS.

The wordlikeness ratings index the probability of judging a string of letters as a real word. In subsequent analyses we computed NLD^1^ (i.e., the number of insertions, deletions, or substitutions required to transform a nonword into a real word, Levenshtein, 1966), N (i.e., the number of words that can be formed by replacing a single letter in an equal-length nonword, Coltheart et al., 1977), and OLD20 (i.e., the mean Levenshtein distance to the 20 closest orthographic neighbors of the nonword, Yarkoni et al., 2008) to examine the contribution of objective measures of wordlikeness to subjective assessments about the probability of a string of letters being a word. We used lexiCAL (Chee et al., 2021) to calculate N and OLD20, as this enables these measures to be computed for pseudowords when a language corpus is supplied as a reference. For this purpose, we used the 244,983 words from the subtitle version of EsPal (Duchon et al., 2013).

The results of the computation of NLDs for each pseudoword condition and its base word are displayed in Table 3. The results of a one-way ANOVA indicated that pseudoword type was significant, *F*(3, 4,796) = 2,941.75, *p* <.001, *η*^2^_p_ =.65. Bonferroni-corrected paired comparisons were all significant, with *p*s equal to or lower than.001. On average, R + S pseudowords most closely resembled their base words, followed closely by R + NonS pseudowords, and then by NonR + S pseudowords. NonR + NonS pseudowords were clearly the most distant from their base words. This index ranges from 0 (no similarity) to 1 (identical strings).

**Table 3:** Mean normalized Levenshtein distance between base words and pseudowords

Type of stimuli	Mean	*SD*	Interval	Range
Words vs. Root + Suffix	.66	.10	[.29,.91]	.62
Words vs. Root + Non-Suffix	.64	.11	[.29,.91]	.62
Words vs. Non-Root + Suffix	.62	.12	[.25,.82]	.57
Words vs. Non-Root + Non-Suffix	.28	.13	[.00,.73]	.73

The mean number of orthographic neighbors for each pseudoword condition is displayed in Table 4. The results of a one-way ANOVA indicated that pseudoword type was significant, *F*(3, 4,796) = 80.22, *p* <.001, *η*^2^_p_ =.05. Bonferroni-corrected paired comparisons were all significant (all *p*s <.001), except for the comparison between R + NonS and NonR + S, which were not significantly different from each other. The R + S condition had the highest average number of orthographic neighbors, followed by the R + NonS and the NonR + S conditions, and then by the NonR + NonS.

**Table 4:** Mean number of orthographic neighbors for each type of pseudoword

Type of stimuli	Mean	*SD*	Interval	Range
Root + Suffix	0.49	0.97	[0, 11]	11
Root + Non-Suffix	0.20	0.57	[0, 6]	6
Non-Root + Suffix	0.22	0.79	[0, 9]	9
Non-Root + Non-Suffix	0.05	0.37	[0, 6]	6

The mean OLD20 values for each pseudoword condition are displayed in Table 5. The results of a one-way ANOVA indicated that pseudoword type was significant, *F*(3, 4,796) = 302.61, *p* <.001, *η*^2^_p_ =.16. As in the case of the number of neighbors, all paired comparisons remained significant after Bonferroni correction (*p*s <.001), with the only exception being the R + NonS versus the NonR + S comparison, for which no significant difference was found. The R + S condition had the lowest mean OLD20, followed by the NonR + S and R + NonS conditions, and then by the NonR + NonS.

**Table 5:** Mean OLD20 for each type of pseudoword

Type of stimuli	Mean	*SD*	Interval	Range
Root + Suffix	2.74	0.57	[1.45, 4.70]	3.25
Root + Non-Suffix	2.95	0.60	[1.50, 4.85]	3.35
Non-Root + Suffix	2.98	0.63	[1.50, 5.00]	3.50
Non-Root + Non-Suffix	3.49	0.75	[1.50, 5.85]	4.35

### Affective dimensions

**Fig. 3:**
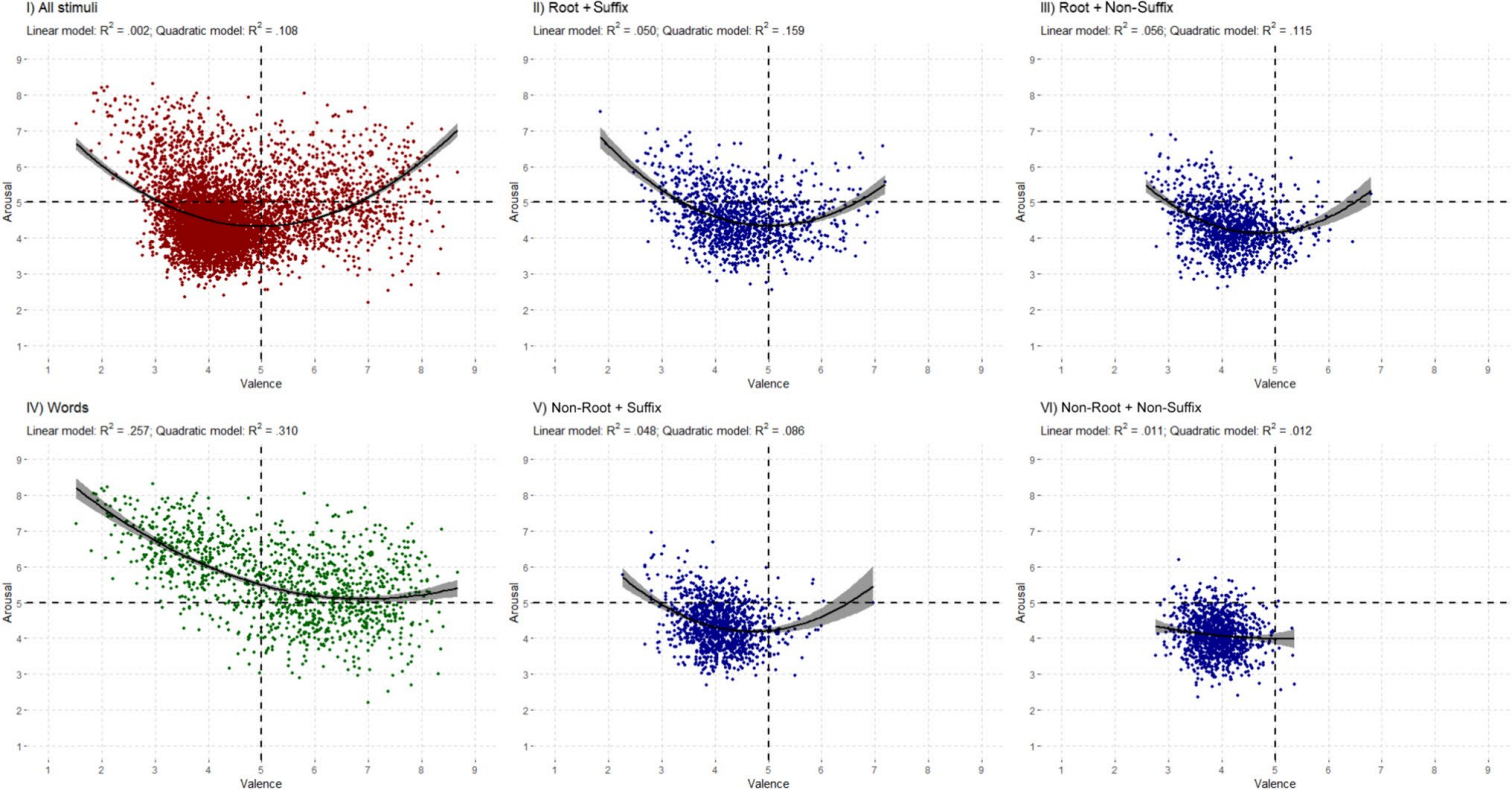
Distribution of the stimuli in the affective space defined by valence and arousal. Valence ratings were plotted against arousal ratings using quadratic regression lines for the full set of stimuli (panel I), the four types of pseudowords (R + S, panel II; R + NonS, panel III; NonR + S, panel V; NonR + NonS, panel VI), and the words (panel IV). The strength of the quadratic relationship was highest for words, decreasing progressively for R + S, R + NonS, and NonR + S pseudowords. The effect was negligible for NonR + NonS

Table 6 displays the mean scores for valence and arousal in each condition. The valence and arousal ratings were analyzed separately for words and pseudowords (see Fig. 3).

**Table 6:** Descriptive statistics for valence and arousal across type of stimuli

Type of stimuli	Mean	*SD*	Interval	Range
Valence				
Words	5.38	1.53	[1.52, 8.67]	7.15
Root + Suffix	4.45	0.82	[1.85, 7.19]	5.33
Root + Non-Suffix	4.18	0.61	[2.58, 6.80]	4.22
Non-Root + Suffix	4.10	0.52	[2.27, 6.96]	4.69
Non-Root + Non-Suffix	3.92	0.39	[2.76, 5.37]	2.61
**Full set**	**4.41**	**1.01**	**[1.52, 8.67]**	**7.15**
Arousal				
Words	5.59	1.09	[2.20, 8.31]	6.11
Root + Suffix	4.59	0.73	[2.56, 7.52]	4.96
Root + Non-Suffix	4.32	0.65	[2.60, 6.88]	4.28
Non-Root + Suffix	4.36	0.63	[2.68, 6.96]	4.28
Non-Root + Non-Suffix	4.08	0.53	[2.36, 6.20]	3.84
**Full set**	**4.59**	**0.92**	**[2.20, 8.31]**	**6.11**

Panel IV shows the relationship between ratings of the two affective dimensions for words. Two regression analyses were carried out, with arousal as the dependent variable and valence as the independent variable. One regression examined a linear relationship, explaining 25.71% of the variance. The other examined a quadratic relationship, which increased the variance explained to 31.02% [*R* =.557, *F*(2, 1,197) = 269.11, *p* <.001]. These results show a U-shaped relationship between valence and arousal, whereby the words with the most extreme valence are also the words with the most extreme arousal.

We carried out the same two types of regression analysis (i.e., investigating linear and quadratic relationships between valence and arousal) on the four types of pseudowords. The quadratic relationship always had more explanatory power than the linear one (see Fig. 3), so we focused on the former.

The model with the highest explanatory power was that of R + S pseudowords: 15.88% of the variance [*R* =.398, *F*(2, 1,197) = 112.985, *p* <.001]. As expected, the model fit is lower than that obtained for words (by almost half), given that these are pseudowords. The next model in terms of explanatory power is that of R + NonS pseudowords, which have an explained variance of 11.52% [*R* =.339, *F*(2, 1,197) = 77.906, *p* <.001]. This was followed by the NonR + S pseudowords, for which the quadratic model explained 8.62% of the variance [*R* =.294, *F*(2, 1,197) = 56.478, *p* <.001]. Finally, as has been consistently observed in all analyses, NonR + NonS pseudowords produced the worst results. Their quadratic model explained only 1.20% of the variance [*R* =.110, *F*(2, 1,197) = 7.292, *p* <.001], suggesting that, while significant, this type of pseudoword appears to have inherited minimal affectivity from its base word.

We also conducted two one-way ANOVAs to compare the valence and arousal ratings of the words and the four types of pseudoword (see Table 6). The difference for valence was significant, *F*(4, 5,995) = 528.92, *p* <.001, *η*^2^_p_ =.26. Bonferroni-corrected paired comparisons revealed that all differences were significant (all *p*s <.001), except for the comparison between R + NonS and NonR + S (*p* =.124). The pattern of results for arousal was the same, that is, a significant main effect, *F*(4, 5,995) = 736.88, *p* <.001, *η*^2^_p_ =.33. All Bonferroni-corrected paired comparisons were significant (all *p*s <.001), except for the comparison between R + NonS and NonR + S (*p* = 1.00).

**Fig. 4:**
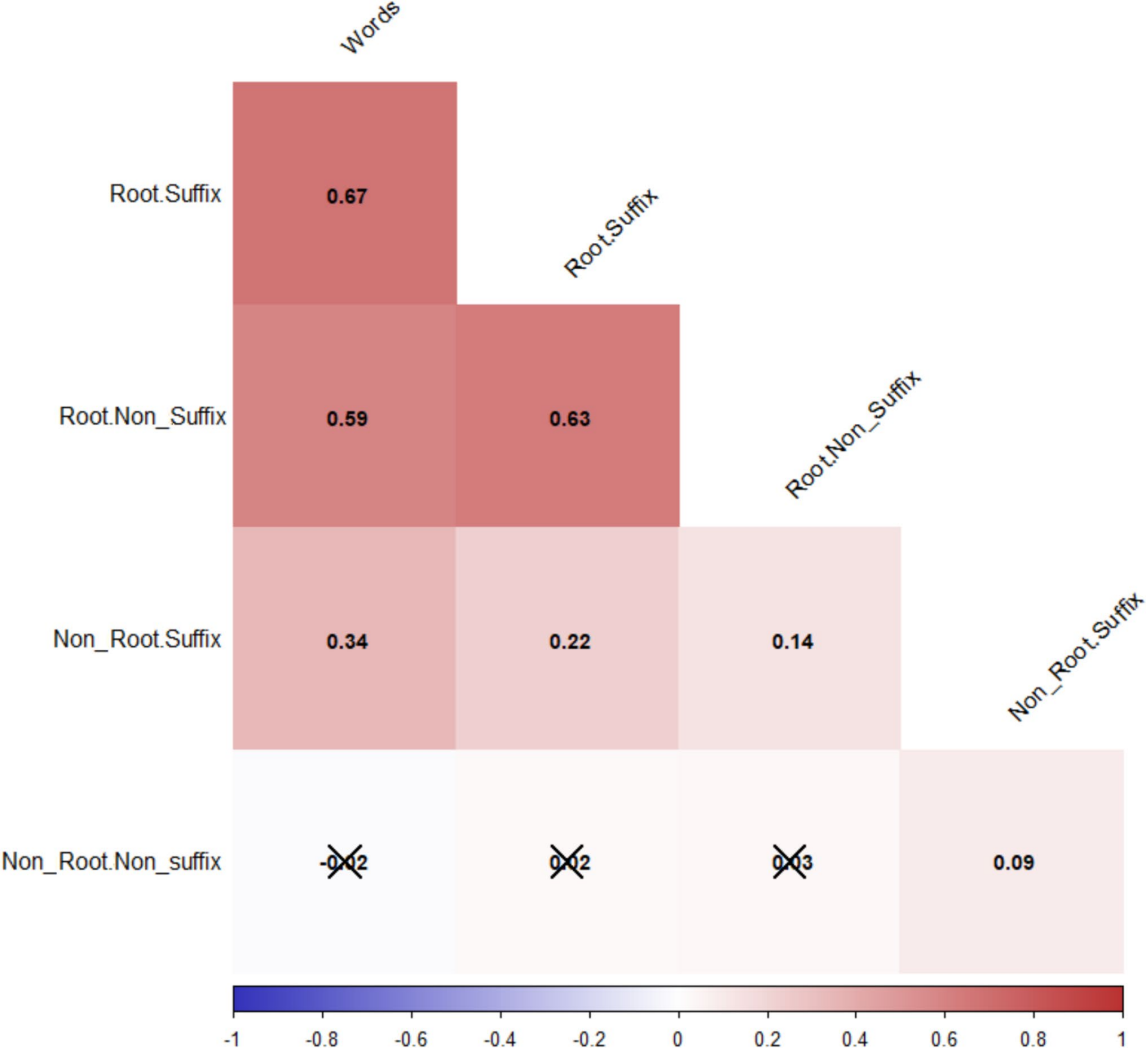
Correlogram showing the valence ratings between the base words and the four types of pseudowords. Crossed out values indicate nonsignificant correlations at a significance level of .05. All *N* = 1,200. The correlations with the valence ratings of the base words were higher for R + S pseudowords. These gradually decreased for R + NonS and NonR + S, becoming nonsignificant for NonR + NonS

In addition to examining the distribution of valence and arousal ratings by word type, as well as the differences between the respective means, we also wanted to investigate the correlation between valence and arousal ratings for words and the four pseudoword types. Regarding valence (see Fig. 4), the most interesting results were found in the correlations with words, where the correlation decreased gradually in the order R + S, R + NonS, NonR + S, and NonR + NonS, with the last becoming nonsignificant. Furthermore, the NonR + NonS type exhibits negligible correlations with the other types of pseudowords.

**Fig. 5:**
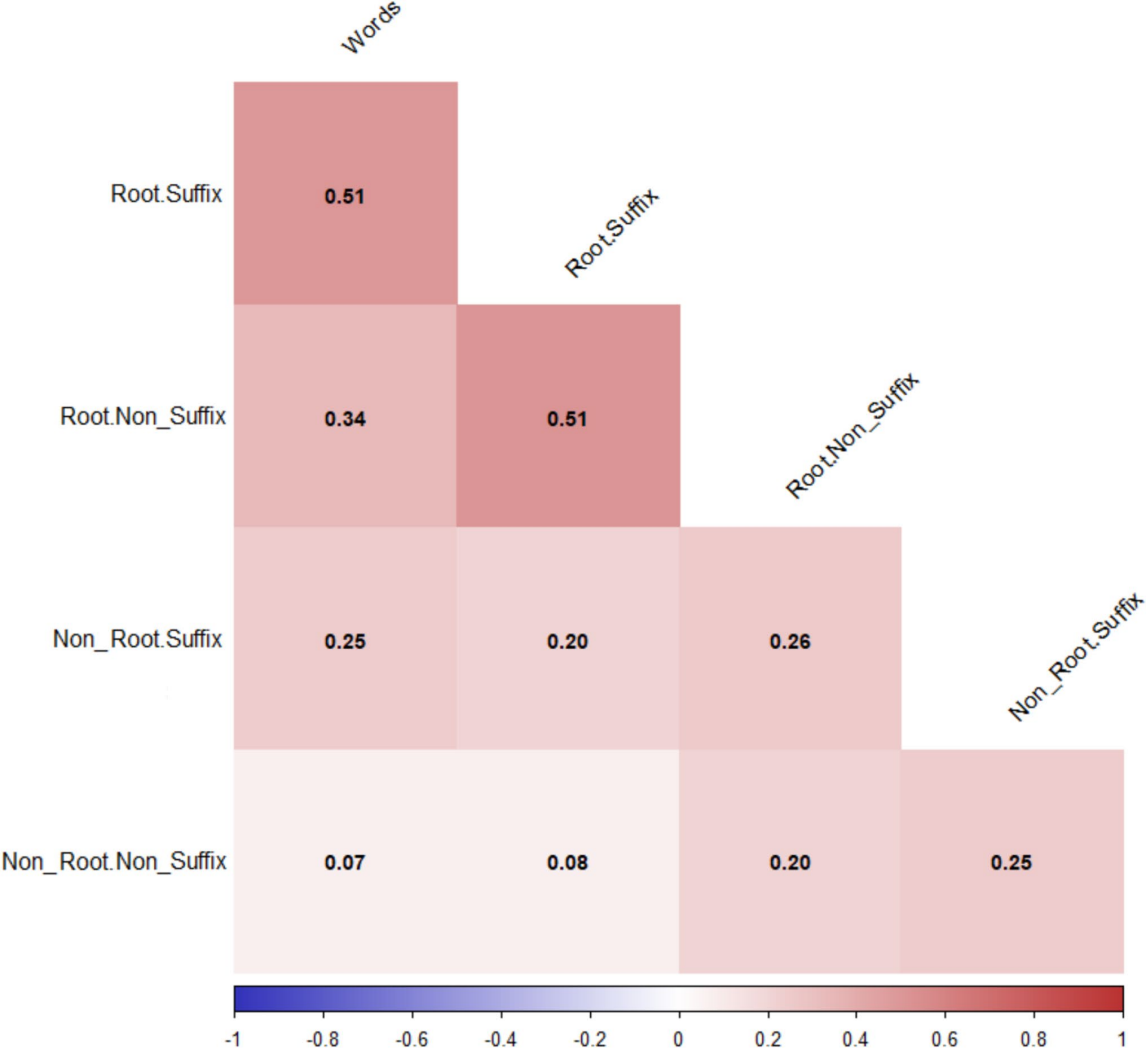
Correlation matrix showing the relationship between the arousal ratings of the base words and the four types of pseudowords. All values significant at a significance level of.05. All *N* = 1,200. Correlations with the arousal score of the base words were higher for R + S pseudowords. These gradually decreased for R + NonS, NonR + S, and NonR + NonS

The pattern is very similar when we focus on arousal (see Fig. 5). However, in general, the correlations are lower than those observed for valence, except for the NonR + NonS pseudowords.

### Effects of morphological composition and affective dimensions on wordlikeness

We also examined how morphological structure and affective dimension scores influence wordlikeness. To this end, we conducted four multiple regression analyses, one for each type of pseudoword. The predictors were the valence and arousal ratings of the pseudowords, the normalized Levenshtein distance (NLD), the orthographic neighborhood size (N), and the OLD20. We also included the frequencies of the roots, the suffixes, and the non-roots and non-suffixes of the pseudowords. We relied on the Zipf values provided in EsPal (Duchon et al., 2013) for these computations. The frequency of roots and non-roots was calculated by the sum of the frequencies of all words that shared the same sequence of letters at the beginning of the genuine word. An analogous procedure was applied to suffixes and non-suffixes, with frequencies computed by aggregating the frequencies of words sharing the same letter sequence at the end of the word. For instance, the frequency of the non-root “tor-” was calculated by summing the frequencies of all words sharing this initial letter sequence, such as toro (bull), tornado (tornado), and tormenta (storm). Similarly, the frequency of the non-suffix sequence “-egio” was obtained by aggregating the frequencies of all words ending with this sequence of letters, such as colegio (school), sortilegio (spell), and privilegio (privilege).

**Fig. 6:**
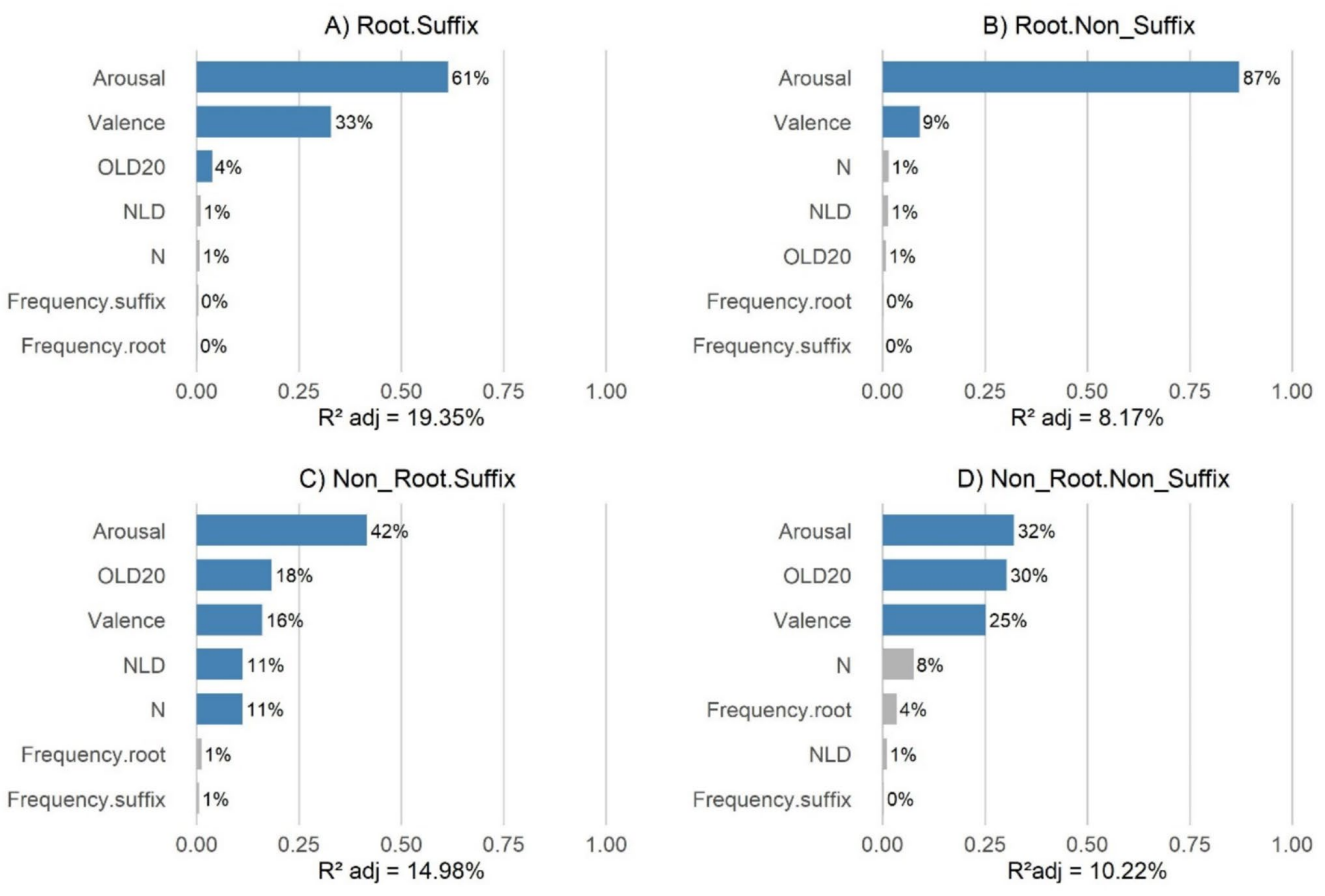
Relative contributions of predictors to the adjusted *R*^2^ in the four models. Each bar represents the proportion of the model’s *R*^2^ explained by a given predictor. Significant predictors are shown in blue, and nonsignificant predictors in gray. In all conditions, the main predictor was arousal

The dependent variable was perceived wordlikeness. In all four analyses, we verified the absence of collinearity among the predictors (all variance inflation factors were below 1.73). Furthermore, in all analyses, all significant predictors had positive effects except for OLD20, which had a negative effect (i.e., lower OLD20 distances indicate higher wordlikeness.). Finally, to evaluate the relative contribution of each predictor, we performed a relative importance analysis (Lindeman et al., 1980) using the *relaimpo* package (Grömping, 2006) in *R*. This method decomposes *R*^2^ into proportions attributable to each predictor while accounting for collinearity and the order of entry into the model. Figure 6 presents the results of the relative importance analysis for each type of pseudoword.

For the R + S pseudowords, the final model explained 19.35% of the variance, *F*(7, 1,192) = 42.11, *p* <.001. The variable that accounted for the largest proportion of variance was arousal (61%), followed by valence, which added an additional 33% to the model. Although OLD20 was also significant, it only accounted for 4% of the variance explained by the final model. For the R + NonS pseudowords, the final model explained less variance (8.4%), *F*(7, 1,192) = 16.24, *p* <.001. The variable that accounted for the highest proportion of the variance in the final model was arousal (87%), followed by valence (9%). Regarding NonR + S pseudowords, the final model explained 14.98% of the variance, *F*(7, 1,192) = 31.19, *p* <.001. Arousal mainly explained this variance (42%), followed by OLD20, valence, NLD, and N, each of which contributed between 11 and 18%. Finally, the model for the NonR + NonS pseudowords explained 10.22% of the variance, *F(*7, 1,192) = 20.49, *p* <.001. This was distributed roughly equally among arousal (32%), OLD20 (30%), and to a slightly lesser extent, valence (25%). From the four analyses it could be concluded that the variable that contributes most to explaining wordlikeness ratings is arousal, followed by valence.

## Discussion

Typically, normative studies have focused on collecting data for several lexicosemantic features of real words. The primary objective of this study was to develop a comprehensive database that provides scores for psycholinguistic features (i.e., wordlikeness) and affective features (i.e., valence and arousal) in a large, carefully controlled set of 4,800 pseudowords with different morphological structures (i.e., two conditions with genuine derivational suffixes, one condition with a part identifiable as a root, and one condition without morphological features). These data were supplemented by ratings for the set of 1,200 Spanish words from which the pseudowords were derived. Our database serves as a resource available to researchers who wish to examine issues such as morphological composition (Crepaldi et al., 2013; Lázaro et al., 2024; Longtin & Meunier, 2005), nonarbitrary relationships between form and meaning (Sidhu & Peetz, 2025; Topolinski et al., 2014), orthographic-to-semantic consistency (Hendrix & Sun, 2021; Yap et al., 2015), the acquisition of conceptual features (Guasch & Ferré, 2021; Mestres-Missé et al., 2014), or the activation of semantic meaning (Gatti et al., 2024; Sulpizio et al., 2021) through the use of novel words (i.e., pseudowords). Furthermore, as we shall see, our analyses highlight the usefulness of pseudowords as a robust methodological tool for investigating the fundamental mechanisms underlying lexical processing, morphological analysis, and affective evaluation.

In terms of reliability, the wordlikeness results showed that participants largely agreed on their estimations of the likelihood of a string of letters being a word. Similarly, there was high agreement across participants in valence scores, while agreement was slightly lower for arousal. Of note, the mean reliability value was not only lower, but there was also greater variation in the range of correlations across participant ratings for arousal in different questionnaires. Also, a high proportion of questionnaires scoring arousal were discarded due to low participant–group correlation (29.73%) compared to those rating wordlikeness (3.23%) and valence (5.96%). Similarly, when we compared scores for words in affective dimensions with those from prior datasets, the validity of our norms was higher for valence than for arousal. This tendency for arousal to be judged less consistently than valence has been observed in several previous normative studies with words (e.g., Guasch et al., 2016; Hinojosa et al., 2016; Monnier & Syssau, 2017; Montefinese et al., 2014). Thus, data from normative studies involving both words and pseudowords align with recent claims that have emphasized the conceptual vagueness of the arousal construct compared to other core emotional aspects, such as valence (Sander, 2025; Smith et al., 2025).

We conducted several analyses to investigate the relationship between the morphological structure of pseudowords and perceived wordlikeness. As expected, wordlikeness scores were very high for real words, averaging 6.60 out of 7, suggesting that some participants considered unknown words to be pseudowords. This is not an unusual finding in a task context in which participants had to rate both words and pseudowords. Interestingly, pseudowords with a genuine suffix were scored as more word-like than pseudowords without morphological structure. Specifically, those composed of a real root and a real suffix (R + S) received the highest ratings, followed by non-root + suffix (NonR + S), root + non-suffix (R + NonS), and non-root + non-suffix (NonR + NonS) pseudowords. Studies involving lexical decision tasks have demonstrated delayed rejection times (Burani et al., 1999; Dawson et al., 2018; Lázaro et al., 2022) and increased peak latencies of pupillary dilation (Lázaro et al., 2023) when presented with pseudowords with a morphological structure that includes roots and affixes, as opposed to those lacking morphological elements. These results point to a role of morphological transparency in determining the plausibility of considering a sequence of letters as a valid lexical entry (Dawson et al., 2018; Lázaro et al., 2022).

The finding that conditions that included real suffixes (i.e., R + S and NonR + S) were perceived as more word-like than those including non-suffixed endings (i.e., R + NonS and NonR + NonS) suggests that these affixes may act as more salient cues than roots when evaluating pseudowords in contexts where suffixes are encountered more frequently than their corresponding roots. This was the case in the current study, which included 1,127 different roots and only 108 different suffixes. Along these lines, previous work has shown that familiarity with suffixes accelerates the acquisition of new words. Specifically, studies using novel word-learning paradigms have found that pseudowords composed of novel roots combined with familiar derivational suffixes (e.g., “clantist” from “clant” + “ist”) are more likely to be recognized as real words in lexical decision tasks after training than those formed from unfamiliar suffixes (Dawson et al., 2021; Tucker et al., 2019). Thus, participants may have been biased towards perceiving NonR + S items as more likely to be real words than R + NonS items due to their overreliance on suffixal cues, despite the latter containing real lexical roots. Nevertheless, these findings should be treated with caution, as they are based not on direct evidence from processing studies, but on participants' subjective estimations. Furthermore, rating genuine words and pseudowords together may have encouraged participants to give inflated wordlikeness ratings to rare words, leading them to rely on superficial cues such as familiar suffixes.

Analyses of measures based on NLD, N, and OLD20 calculations confirmed that the R + S pseudowords were those that more closely resembled real words. However, they also revealed that R + NonS pseudowords were closer to real words than NonR + S pseudowords (NLD), or that there were no differences between R + S and NonR + S pseudowords (N and OLD20). This distinct pattern of results suggests that objective measures do not fully reflect perceived wordlikeness across pseudoword conditions with different morphological structures. Typically, subjective ratings and objective measures of word similarity such as phonotactic probability or lexical neighborhoods exhibit a strong positive correlation (Aljasser et al., 2025; Bailey & Hahn, 2001; Frisch et al., 2000). This indicates that human listeners are sensitive to statistical regularities in their language, such as phonotactic probabilities and similarity to existing words, and utilize this implicit knowledge when forming subjective opinions. However, the correlation is not perfect, suggesting that each measure captures a unique aspect of wordlikeness (Bailey & Hahn, 2001). We will return to the distinction between objective and subjective measures when discussing the contribution of different variables to perceived wordlikeness.

With regard to the affective variables, the results of regression and correlational analyses showed a consistent pattern of results. The relationship between valence and arousal ratings of words showed the typical U-shaped distribution (Ćoso et al., 2019; Guasch et al., 2016; Hinojosa et al., 2016; Monnier & Syssau, 2014; Montefinese et al., 2014; Soares et al., 2012; VõM et al., 2009). This finding suggests that words at the extremes of the valence continuum (i.e., positive and negative words) are associated with higher arousal scores. It is worth noting that the results of the regression analyses for pseudowords indicated that the model for R + S pseudowords had the greatest explanatory power, followed by the models for R + NonS, NonR + S, and NonR + NonS pseudowords. The results of the analyses of mean valence and arousal scores showed a similar pattern, with R + S pseudowords producing scores closer to those of words in both dimensions. However, there was no significant difference between R + NonS and NonR + S pseudowords. Additionally, correlations between valence and arousal ratings for words and pseudowords with different morphological composition were higher for valence than for arousal. Furthermore, these correlations decreased gradually, with the highest correlation observed for R + S pseudowords, followed by R + NonS and then NonR + S pseudowords. These correlations were not significant for NonR + NonS pseudowords.

Overall, the results of these analyses align with previous observations that pseudowords express emotional meaning (Gatti et al., 2023; Martínez-Tomás et al., submitted; Sulpizio et al., 2021). However, the variance explained by valence and arousal ratings is only modest, and the effects diminish with reduced morphological transparency. This suggests that our findings should be interpreted with caution. In this line we observed that the emotional connotations (i.e., valence and arousal) of pseudowords, as well as the U-shaped relationship between valence and arousal, become considerably weaker as their morphological similarity to the base word decreases. These results show that the morphological composition of words influences how they are perceived in terms of valence and arousal. They also suggest that affective meaning can be communicated through pseudowords, at least to some extent (Hinojosa et al., 2022; Kuperman et al., 2014; Majid, 2012; Ponsonnet, 2018). Notably, while R + S pseudowords received the highest ratings in both affective dimensions, R + NonS items received the second-highest ratings. This contradicts our findings regarding wordlikeness, which indicated that suffixation played a role in perceiving the similarity of a sequence of letters as a word. In contrast, conveying affective meaning appears to rely more heavily on roots, which are the core meaningful elements of words. Therefore, our data suggest that the representation of affective features is partially activated by roots, even in the absence of real bounded morphemes.

Finally, the analyses using multiple regression models revealed that arousal and valence were the main predictors of wordlikeness. Once again, the relationship between these variables was more evident in pseudoword conditions including roots (i.e., R + S pseudowords, followed by R + NonS, NonR + S, and finally NonR + NonS pseudowords). These results suggest that participants perceived that sequences of letters derived from positive words or highly arousing words had a higher plausibility of being a real word relative to the lexicon as a whole. As mentioned previously, the effects of wordlikeness are heavily influenced by phonotactic and orthographic constraints (Bailey & Hahn, 2001; Frisch et al., 2000; Vitevitch & Luce, 1999). Notably, previous studies have demonstrated the existence of statistical regularities in the phonotactic and orthographic features of words and pseudowords conveying affective meaning across languages. These nonarbitrary form-to-affective meaning associations seem particularly evident in words with high-arousing conceptual referents. Along these lines, high-arousing words and pseudowords tend to include more complex consonant clusters and have a higher pitch (Aryani et al., 2018a; Aryani et al., 2018b; Schmidtke & Conrad, 2024). Sibilant fricatives and voiceless consonants (e.g., /s/,/z/,/ʃ/) also seem to be overrepresented in high-arousing words, as these phonemes imitate the hissing sounds made by threatening animals such as snakes (Calvillo-Torres et al., 2025; Conrad et al., 2022; de Zubicaray & Hinojosa, 2024). Similar connections between form and meaning have been observed in positive words. For example, front vowels (e.g., /i/and/i:/) seem to be associated with positive meaning, possibly because the muscles used to articulate these sounds overlap with those involved in smiling (Körner & Rummer, 2022; Sidhu et al., 2022; Yu et al., 2021). Additionally, initial bilabial (e.g., /p/,/b/) or velar phonemes (e.g., /g/,/ŋ/), as well as final labiodental phonemes (e.g., /v/), are overrepresented in positive words (de Zubicaray et al., 2023). Furthermore, certain morphological markers, such as those used to express diminution, have been found to carry positive meanings (Hinojosa et al., 2022). Consequently, participants' sensitivity to these surface-level cues may account for the observation that pseudowords derived from words with high valence and arousal scores were more likely to be judged as genuine words.

The results of our correlational analyses showed that arousal and, to a lesser extent, valence predicted wordlikeness in pseudowords with genuine roots (in both real suffix and non-suffix conditions), whereas the effects of OLD20, NLD, and N were negligible. However, while arousal remained the primary contributor to perceived wordlikeness in pseudowords without real roots, OLD20 accounted for nearly the same amount of variance. These findings suggest that measures based on the transformation of one representation (e.g., a pseudoword) into another (e.g., the distance to neighboring words, as in NLD) or to words throughout the entire lexicon (as in OLD20 or N) do not solely determine people’s intuitions about whether a sequence of letters is likely to be a word (Bailey & Hahn, 2001). It is worth noting that wordlikeness rating tasks are thought to be mainly dominated by estimations about the probability with which particular sequences of sounds occur, whereas the similarity to real words is less relevant. In contrast, objective measures such as OLD20 or the number of orthographic neighbors are mainly concerned with the similarity of a string of letters to existing words from the mental lexicon (Bailey & Hahn, 2001; Do & Lai, 2020; Frisch et al., 2000; Vitevitch & Donoso, 2012). Also, measures such as NDL capture derivational proximity to a single the base word. Therefore, they may not fully reflect the type of lexicon-level plausibility that participants are likely to consider when making probability judgments of wordlikeness. Previous work suggests that subjective wordlikeness is sensitive to broader distributional properties of the lexicon such as phonotactic and orthographic sequence typicality or lexical neighborhood structure (Bailey & Hahn, 2001; Woollams et al., 2011), which are not directly captured by single-anchor distance measures. From this perspective, the weak association between subjective wordlikeness ratings and NLD may reflect a difference between the similarity space indexed by this metric and the construct targeted by our task, rather than indicating that formal similarity plays a limited role in wordlikeness judgments.

In our view, the results of the regression analyses suggest that when pseudowords contain real roots that provide strong cues about frequently encountered sequences of phonemes, information from the activation of lexical neighbors becomes less relevant when assessing perceived wordlikeness. Conversely, when pseudowords do not include roots, participants may rely more heavily on the activation of potential lexical candidates to evaluate wordlikeness, since the cues about sequence typicality provided by the roots are less salient. One way to test some of these predictions would be to collect ratings from pseudowords that include morphologically simple words as roots, which should provide even more salient phonotactic cues. This should reduce the contribution of objective measures to estimations of wordlikeness relative to pseudowords with real roots that are not genuine words.

Alongside their theoretical implications, the current findings highlight the importance of selecting pseudowords carefully when designing experiments. As Rastle et al. (2002) pointed out, researchers must ensure that there are no systematic differences between words and pseudowords, especially in studies where participants might become aware of structural biases in the stimuli. For instance, in studies where a significant proportion of the pseudowords contain a particular suffix (e.g., “-ist”), participants may recognize this as a pattern, as opposed to studies where a variety of suffixes are used to create pseudowords. This could result in increased morpheme interference in lexical decision tasks (i.e., participants may become slower and less accurate at rejecting pseudowords comprising frequently encountered existing morphemes) and stronger morphological priming effects, potentially skewing the results (Beyersmann et al., 2020; Crepaldi et al., 2010; Lázaro et al., 2024; Rastle et al., 2004). Another important question concerns the measurement of wordlikeness. In line with prior observations (Bailey & Hahn, 2001; Do & Lai, 2020), our findings suggest that objective calculations and the perceived probability of a nonword being a word account for different aspects of wordlikeness, such as the similarity of a nonword to closely related lexical items or phonotactic probability. These aspects should be carefully considered when designing experiments aimed at investigating the determinants of wordlikeness.

This study is not without its limitations, which also open up avenues for future research and the extension of our norms. This normative study does not furnish behavioral or neurocognitive evidence to ascertain whether the pseudowords predict morphological or affective processing. Data from future studies involving lexical decision, categorization or naming tasks, and the collection of reaction times, eye tracking, or electroencephalographic measures will demonstrate the value of this dataset. They could also test assumptions regarding the influence of suffixes and the role of objective computations in subjective estimations of the wordlikeness of pseudowords with different morphological compositions. Another point that deserves attention is the fact that Spanish is spoken in many different countries and in a variety of sociolinguistic settings, often alongside other languages. This diversity and cross-linguistic exposure may have influenced how the participants perceived the pseudowords, particularly in terms of their similarity to real words. Although our sample primarily consisted of participants born in Spain, data from cross-dialectal replications or studies involving bilingual participants would be valuable for validating and expanding this dataset. Furthermore, future normative studies could consider generating pseudowords from prefixed base words. This could be useful for exploring the contribution of affixes and suffixes to word recognition, as some studies have reported processing differences between these types of affixes (Beyersmann et al., 2015; Giraudo et al., 2025).

## Conclusion

Studies involving pseudowords have greatly enhanced our understanding of the role of orthographic, morphological, and phonological elements in word processing (Martínez-Tomás et al., 2025). In recent years, research has expanded to address aspects such as semantics and syntax. However, there is still a lack of normative studies that provide pseudowords with well-controlled structures and assess them against different lexicosemantic variables. Here, we present wordlikeness, valence, and arousal scores for 4,800 Spanish pseudowords (and 1,200 real words) that were formed by altering morphological components (roots and suffixes). We hope that these norms will facilitate research in language and affective sciences. Furthermore, the database paves the way for cross-linguistic research by providing a model for the development of similar resources in other languages.

## Supplementary Information

Below is the link to the electronic supplementary material.

Supplementary file1 (PDF 71.4 KB)

## Data Availability

Data used in the analysis are available at: https://osf.io/baues/. This study was not preregistered.

## Footnotes

1 The Levenshtein distance is a value that can be interpreted differently depending on the length of the strings being compared. For example, a difference of one letter between two two-letter strings is not the same as a difference of one letter between 10-letter strings. To avoid this issue, the normalized Levenshtein distance can be computed. This is calculated by dividing the Levenshtein distance by the length of the longest string in the comparison and subtracting the result from 1.

## Code availability

The code is available at: https://osf.io/baues/.

## Declarations

### Open practices statement

Data are available at https://osf.io/baues/. The study was not preregistered.
