# Supplementary material for: When the meaningless make sense: Wordlikeness and affective norms for 4,800 pseudowords and 1,200 Spanish words
Source: Behav Res Methods. 2026 Apr 1;58(4):103. doi: 10.3758/s13428-026-02976-4 (PMC13043531; doi:10.3758/s13428-026-02976-4)
Supplement: Supplementary file 1 — Supplementary file1 (PDF 71.4 KB) [file 13428_2026_2976_MOESM1_ESM.pdf]

Appendix

Instructions in Spanish and their corresponding English translations

Instructions in Spanish

Instrucciones de valencia

A continuación, te vamos a presentar una serie de secuencias de letras. Algunas son palabras reales en castellano, mientras que otras son inventadas. Te pedimos que, por favor, nos indiques el grado de felicidad o infelicidad que sientes al leerlas. Para ello vas a usar una escala de 9 puntos donde 1 = muy infeliz y 9 = muy feliz. Aunque no conozcas el significado de estas secuencias de letras o te resulten extrañas, no pasa nada. No dediques demasiado tiempo a reflexionar sobre ellas, valóralas basándote en tu primera impresión. No hay respuestas buenas ni malas. Recuerda que puedes usar todos los valores de la escala.

Instrucciones de activación

A continuación, te vamos a presentar una serie de secuencias de letras. Algunas son palabras reales en castellano, mientras que otras son inventadas. Te pedimos que, por favor, nos indiques el grado de activación que sientes al leerlas. Para ello vas a usar una escala de 9 puntos donde 1 = muy tranquilo y 9 = muy activado. Aunque no conozcas el significado de estas secuencias de letras o te resulten extrañas, no pasa nada. No dediques demasiado tiempo a reflexionar sobre ellas, valóralas basándote en tu primera impresión. No hay respuestas buenas ni malas. Recuerda que puedes usar todos los valores de la escala.

Instrucciones de wordlikeness (semejanza con palabra)

A continuación, te vamos a presentar una serie de secuencias de letras. Algunas son palabras reales en castellano, mientras que otras son inventadas. Te pedimos que, por favor, nos indiques la probabilidad de que estas secuencias pudieran ser una palabra en

For Review Only

castellano. Para ello vas a usar una escala de 7 puntos donde 1 = muy poco probable que esto pudiera ser una palabra en castellano y 7 = muy probable que esto pudiera ser una palabra en castellano. Aunque no conozcas el significado de estas secuencias de letras o te resulten extrañas, no pasa nada. No dediques demasiado tiempo a reflexionar sobre ellas, valóralas basándote en tu primera impresión. No hay respuestas buenas ni malas. Recuerda que puedes usar todos los valores de la escala.

Instructions in English (translations from Spanish)

Valence instructions

Next you will see a series of sequences of letters. Some of them are real words in Spanish, while others are invented. We ask you to rate the degree of happiness and unhappiness you feel when reading them. To assess the words, you should give your rating on a scale of 1 = very unhappy and 9 = very happy. Even if you don't know the meaning of these letter sequences or if they seem strange to you, that's okay. Don't spend too much time thinking about them, just evaluate them based on your first impression. There are no right or wrong answers. Remember that you can use all the values of the scale.

Arousal instructions

Next you will see a series of sequences of letters. Some of them are real words in Spanish, while others are invented. We ask you to rate the degree of activation you feel when reading them. To assess the words, you should give your rating on a scale of 1 = very calm and 9 = very activated. Even if you don't know the meaning of these letter sequences or if they seem strange to you, that's okay. Don't spend too much time thinking about them, just evaluate them based on your first impression. There are no right or wrong answers. Remember that you can use all the values of the scale.

Wordlikeness instructions

For Review Only

Next you will see a series of sequences of letters. Some of them are real words in Spanish, while others are invented. We ask you to rate the probability that these sequences could be a word in Spanish. To assess the words, you should give your rating on a scale of 1 = very improbable this could be a word in Spanish and 9 = very probable this could be a word in Spanish. Even if you don't know the meaning of these letter sequences or if they seem strange to you, that's okay. Don't spend too much time thinking about them, just evaluate them based on your first impression. There are no right or wrong answers. Remember that you can use all the values of the scale.

For Review Only
